# Nursing Student Led Blood Pressure Education in a Community Mental Health Setting: A Pre Post Test Study

**DOI:** 10.1111/inm.70385

**Published:** 2026-10-30

**Authors:** Catherine Stephen, Taylor Yousiph, Elizabeth Halcomb, Elissa‐Kate Jay, Brandon Smith, Christopher Patterson, Elizabeth Curtis, Judy Mullan, Lorna Moxham

**Affiliations:** ^1^ School of Nursing, Faculty of Science Medicine and Health University of Wollongong Wollongong Australia; ^2^ Graduate School of Medicine, Faculty of Science Medicine and Health University of Wollongong Wollongong Australia

**Keywords:** health education, health literacy, hypertension, mental illness, nursing student

## Abstract

People living with mental illness have greater cardiovascular risk, more lifestyle risk factors and lower health literacy than the general population. The high prevalence of hypertension among people living with mental illness places them at a significantly higher risk of premature morbidity and mortality. Identifying hypertension and empowering consumers to understand its significance are key to controlling elevated blood pressure. Student nurse led blood pressure education in community mental health settings may improve hypertension awareness while building nursing students' confidence and competence. This study sought to evaluate the impact of a preregistration nursing student led blood pressure education activity on consumer blood pressure knowledge and nursing students' health education competence. A single group pre post test intervention study was undertaken during a community mental health clinical placement (Recovery Camp, New South Wales, Australia). Nursing students delivered a brief blood pressure education activity to consumers living with mental illness. Consumer knowledge was assessed using the Hypertension Evaluation of Lifestyle and Management (HELM) scale, and student competence was assessed using the Nurse Health Education Competence Instrument (I‐CEpSE). Twenty‐one consumers completed paired assessments. Mean HELM scores increased from 5.0 (SD 2.65) pre activity to 6.9 (SD 2.69) post activity (*p* < 0.001). Forty‐four students completed paired assessments, with I‐CEpSE total scores increasing from 210.95 (SD 28.81) to 257.27 (SD 30.22) (*p* < 0.001). This study has demonstrated that a structured, student led blood pressure education activity embedded within a community mental health placement improved both consumer hypertension knowledge and student reported health education competence.

## 1 Introduction

Over 1.1 billion people internationally are living with mental illness (World Health Organization 2019). People living with mental illness experience substantially poorer physical health outcomes (Zabeen et al. 2021) and have a life expectancy some 10–20 years less than the general population (Australian Institute of Health and Welfare 2025). In particular, compared with the general population, people living with mental illness have higher rates of cardiovascular morbidity and mortality (Zabeen et al. 2021). This is linked to the greater number of lifestyle‐risk factors, including poor diet, limited exercise alcohol and substance use disorders, all of which increase the prevalence of chronic conditions such as cardiovascular disease (O'Connor et al. 2023). Indeed, among people living with mental illness, cardiovascular disease is the most common cause of death, with hypertension identified as a main risk factor (Shahimi et al. 2022). Given the high prevalence and impact of hypertension on those living with mental illness, this is an important target group for intervention.

## 2 Background

Globally, elevated blood pressure contributes substantially to morbidity and mortality and is responsible for a significant proportion of preventable cardiovascular events (Zhou et al. 2021). Hypertension is challenging to both identify and treat. As high blood pressure is usually symptomless, people often only become aware that they have hypertension on routine health screening or when complications develop. Even when people are aware that they have hypertension, many continue to have uncontrolled blood pressure and increased cardiovascular risk (Schutte et al. 2022). It is difficult to achieve blood pressure control, as this requires a combination of lifestyle risk reduction and daily medication to effectively manage. This demonstrates that improving health literacy and empowering people to understand and act on blood pressure information are a key strategies to reduce the hypertension burden (Schutte et al. 2022; Wang et al. 2025).

Helping people to understand their blood pressure readings and the associated cardiovascular risk is an important strategy for improving both help‐seeking behaviours and long‐term health outcomes (Schutte et al. 2024; Unger et al. 2020). Limited health literacy has been associated with poorer mental health status (Haeri‐Mehrizi et al. 2024), and those living with mental illness generally have poorer health literacy than the general population (Degan et al. 2021). Health literacy interventions such as patient education programmes that support individuals to interpret health information and engage in self‐management behaviours have been shown to improve blood pressure control and cardiovascular risk management (Beyna et al. 2026).

Health education is a core component of the nursing role, particularly in primary and community health settings, where prevention and chronic disease management are central to care delivery. Therefore, it is vital that nursing students are prepared to deliver effective health education on blood pressure and self‐management as part of their preregistration education. Clinical placements provide important opportunities for students to develop competence in health promotion and patient education as they enable students to translate theoretical knowledge into practice and develop practical skills in real‐world care settings (Moxham et al. 2025). However, the extent to which preregistration nursing curricula consistently prepare students to deliver structured blood pressure education remains unclear (Halcomb et al. 2020). In particular, little is known about whether community based clinical placements provide structured opportunities for students to develop health education competence in real‐world settings (Bulfone et al. 2024).

### 2.1 Aim

The aim of this paper is to evaluate the impact of a student nurse led blood pressure education activity (Heart Sense) delivered within a community mental health clinical placement on consumer blood pressure knowledge and nursing students' health education competence.

## 3 Methods

A single‐group pre‐post design was implemented to assess the effectiveness of the Heart Sense intervention by evaluating outcomes before and after the intervention.

### 3.1 Setting

Recovery Camp is a non‐traditional mental health placement that offers preregistration nursing students immersive, experiential learning with people who have lived experience of mental illness (‘consumers’). Grounded in therapeutic recreation and recovery oriented practice, the setting facilitates strengths‐based, person‐centred engagement through a programme of activities attended by students, consumers and registered nurse facilitators (Moxham et al. 2024). This context provided a practical platform for Heart Sense, enabling nursing students to deliver blood pressure education embedded in everyday interactions and aligned with holistic, recovery focused care.

### 3.2 Intervention

Heart Sense is a structured, student nurse led blood pressure awareness and education activity. The activity was informed by previous primary care nurse led interventions to improve blood pressure control (Authors Own). The preregistration nursing students completed a two hour training session the day before the Heart Sense activity. This session covered fundamental theoretical content, including the burden and impacts of high blood pressure, standardised automated blood pressure measurement techniques, documentation and therapeutic communication. Additionally, this session incorporated practical skills training in which students practiced blood pressure measurement techniques on each other under supervision.

During the Heart Sense activity, each consumer was paired with two nursing students. Students obtained an automated blood pressure reading and delivered brief, plain language health education supported by publicly available visual resources from national and international cardiovascular and hypertension organisations (Heart Foundation 2025; International Society of Hypertension 2026). Interactions lasted approximately 20 min, with additional time allocated as needed for consumer led discussion. The activity sought to enhance consumer knowledge rather than being a diagnostic assessment or the provision of medical advice. To ensure appropriate clinical oversight of incidental findings, students used a decision making guide that prompted repeat measurement and prompt referral to the supervising registered nurse if blood pressure readings met predefined escalation criteria.

### 3.3 Participants and Recruitment

Consumer participants were drawn from adults living with mental illness attending a single Recovery Camp programme who were willing to participate in the blood pressure education activity. Consumers were excluded if they were unable to provide informed consent or were unable to participate due to acute distress or other clinical considerations identified by Recovery Camp staff.

Nursing student participants were eligible to participate if they were enrolled in any year of a preregistration nursing programme and attending a clinical placement at Recovery Camp. Students who did not attend the preparatory session were not eligible to participate in the intervention delivery.

Eligible participants were provided with a brief verbal and written explanation of the study and the opportunity to ask any questions before they indicated whether they would like to participate. Written informed consent was obtained prior to participation.

### 3.4 Data Collection

Data were collected in October 2025 by two researchers (E.J., C.S.) at two time points: (1) pre activity, scheduled at the commencement of Recovery Camp, and (2) post activity, collected after completion of the blood pressure awareness and education activity. Demographic data were collected at baseline only.

Consumer knowledge was assessed using the Hypertension Evaluation of Lifestyle and Management (HELM) knowledge scale (Schapira et al. 2012). HELM is a 14‐item tool comprising true/false and multiple choice questions, written at a low to medium literacy level, designed to assess knowledge of hypertension, lifestyle factors and management. As HELM was designed and tested in America, minor wording changes were made to ensure relevance to an Australian context (e.g., ‘ounces’ of beer was converted to ‘millilitres’ of beer).

Student competence was assessed using the Nurse Health Education Competence Instrument (I‐CEpSE) (Pueyo‐Garrigues et al. 2022). The I‐CEpSE comprises 58 items across three domains: cognitive, psychomotor and attitudinal/affective. The cognitive domain measures respondents' knowledge of health determinants and health education, including pedagogical resources and educational techniques. These 12 items are rated on a 5 point Likert scale from 1 (very low) to 5 (very high). The psychomotor domain includes 26 items that measure social and educational skills related to health education practice on a 5 point Likert scale from 1 (completely disagree) to 5 (completely agree). The attitudinal and affective domain measures respondents' personal qualities related to health education and practice, including their intentions, attitudes and confidence. This domain includes nine items, rated on a 5‐point Likert scale from 1 (completely disagree) to 5 (completely agree). Higher scores indicate greater self‐reported knowledge, stronger perceived skills and more positive attitudes towards delivering health education. The total possible scores range from 0 to 290. Given that the Cronbach alpha for each domain and the whole tool is > 0.810, this demonstrates high internal consistency (Pueyo‐Garrigues et al. 2021).

### 3.5 Data Analysis

Data were collected in hard copy using paper forms and entered into a Microsoft Excel spreadsheet by the researchers. Data were checked for completeness and accuracy by two authors (E.J., B.S.). Responses to the HELM scale were recoded as correct (1) or incorrect (0), producing a possible total score range of 0 to 14. Descriptive statistics were calculated to summarise participant characteristics and scale scores. Paired *t*‐tests were used to compare pre and post activity outcomes. A *p* value < 0.05 was considered statistically significant.

### 3.6 Ethical Approval and Informed Consent

This study received approval from the University of Wollongong HREC (Approval number H2025‐1462). All potential participants were informed that participation was voluntary and that choosing not to participate would not affect their involvement in the programme or their placement.

## 4 Results

### 4.1 Participant Characteristics

While 27 consumers agreed to participate, six (22.2%) did not complete either the pre or post test, resulting in a final sample of 21 consumers. The mean age of consumer participants was 50.6 years (SD 12.95, range 28–68 years) and 11 (52.4%) were male. Consumer participants had varying diagnoses, including schizophrenia (*n* = 7, 33.3%), depression (*n* = 8, 38.1%) and anxiety (*n* = 7, 33.3%). Just over half of the consumer participants reported multiple mental health diagnoses (*n* = 11, 52.4%) and ongoing physical health issues (*n* = 11; 52.4%). When asked how frequently they visited their general practitioner, nine (42.9%) consumer participants reported attending monthly. Only eight (38.1%) participants reported having their blood pressure measured every visit and five (23.8%) participants indicated that their blood pressure was never measured or only when they requested it.

Most (*n* = 36; 81.8%) of the 44 student participants were in the second year of their preregistration nursing programme. The mean age of student participants was 31.98 years (SD 11.66; range 19–55 years). For almost all (*n* = 40; 90.9%) of the student participants, this was their first mental health clinical placement. However, all students reported having learned about the risks of high blood pressure in their course.

### 4.2 Consumer Knowledge

Consumer participants' pre and post activity scores are provided in Table 1. The mean HELM score increased from 5.00 (SD 2.65) pre activity, with individual scores ranging from 0 to 12, to 6.90 (SD 2.69) post activity, with individual scores ranging from 2 to 12. A paired samples *t*‐test revealed a statistically significant improvement in consumer knowledge following the blood pressure awareness and education activity (*t*(20) = 4.48, *p* < 0.001). Overall, 16 (71.4%) of consumer participants achieved a higher post activity score.

**TABLE 1:** Consumer participants' pre and post activity HELM Scale scores.

Participant ID	Pre activity	Post activity
P01	5	4
P02	5	4
P03	3	3
P04	5	5
P05	12	12
P06	7	7
P07	5	6
P08	1	2
P09	3	4
P10	3	5
P11	8	10
P12	4	6
P13	7	9
P14	8	10
P15	8	11
P16	6	9
P17	4	8
P18	3	7
P19	5	9
P20	3	8
P21	0	6

A review of responses to individual HELM items showed that the lowest scoring items changed from pre to post test. Pre activity, the lowest scoring items, were related to factors that increase hypertension risk (*n* = 1, 4.8%), lifestyle changes most likely to impact blood pressure (*n* = 1, 4.8%), and optimal timing of blood pressure medication (*n* = 3, 14.2%). Post activity, the lowest scoring items related to factors that increase hypertension risk and the potential consequences of uncontrolled hypertension (*n* = 4; 19%) and consumers' understanding of home blood pressure measurement procedures (*n* = 3; 14.3%).

Conversely, several items were answered correctly by a greater proportion of consumer participants. Pre activity, the highest scoring items were related to blood pressure criteria, the need for medication in addition to exercise, and identifying calorie‐dense foods, each with 12 (57.1%) correct responses. Post activity, the highest scoring items were variability in blood pressure measurement (*n* = 18, 85.7%), while 15 (71.4%) correctly answered items about blood pressure criteria, the need for medication in addition to exercise and dietary changes that lower blood pressure. Although no consumer participant answered all HELM scale items correctly and no individual item was answered correctly by all participants, the overall pattern suggests that a single blood pressure awareness and education activity was associated with measurable improvements in consumer knowledge.

### 4.3 Nursing Student Competence

As shown in Table 2, statistically significant improvements were observed across all three I‐CEpSE domains following students' participation in the blood pressure awareness and education activity (*p* < 0.001). The largest mean increase occurred in the cognitive domain, followed by the psychomotor domain and the attitudinal and affective domain. Mean total I‐CEpSE scores increased from 211.62 (SD 29.47, range 98–270) pre activity to 259.52 (SD 28.39, range 177–290) post activity. A paired samples *t*‐test confirmed that this change was statistically significant (*t*(41) = 9.91, *p* < 0.001).

**TABLE 2:** Student participants' mean pre and post activity I‐CEpSE scores.

Domain	Pre activity		Post activity		Pre post comparison	
	Mean (SD)	Range	Mean (SD)	Range	*t*(41)	Cohen's *d* (95% CI)
Cognitive	78.21 (12.03)	29–102	102.33 (12.21)	71–115	11.26*	1.74 (1.25–2.21)
Psychomotor	97.90 (16.30)	47–130	116.36 (13.06)	78–130	6.87*	1.06 (0.68–1.44)
Attitudinal and affective	34.55 (5.86)	22–45	40.83 (5.02)	25–45	8.12*	1.25 (0.84–1.65)
Total score	210.67 (28.56)	98–270	259.52 (28.39)	177–290	10.41*	1.61 (1.14–2.06)

* *p* < 0.001.

## 5 Discussion

This study investigated the impact of a nursing student led blood pressure education activity delivered within a community mental health clinical placement on consumer blood pressure knowledge and nursing students' health education competence. Consumer hypertension knowledge significantly improved following the intervention, and students demonstrated significant gains across health education knowledge, skills and attitudes. These findings suggest that structured, supervised blood pressure education activities can be integrated into mental health placements in ways that support both consumer learning and the development of student competence and confidence.

Limited knowledge of hypertension negatively influences health behaviours and practices (Singh et al. 2020). There is increasing recognition of the need to strengthen prevention strategies to address cardiovascular risk among people living with mental illness (Palmer et al. 2018). Blood pressure education initiatives are a key component in addressing the poorer physical health outcomes experienced by people living with mental illness. Initiatives like Heart Sense, which build an understanding of blood pressure, can strengthen health literacy and support engagement in hypertension self‐management. Health education is a first‐line strategy to control hypertension (Williams et al. 2018), and promotes patient empowerment (Zhao et al. 2020). Empowerment is a central tenet of mental health recovery (Leamy et al. 2011), with empowerment‐based approaches supporting independence, social inclusion and confidence (Shah and Goldin 2026).

Findings from this study demonstrated that Heart Sense increased consumer knowledge about hypertension. This finding resonates with previous health education interventions directed towards people living with mental illness which have demonstrated positive outcomes. For example, a 12‐week diabetes education programme reported by Fenton et al. (2021), found improvements in consumer self‐knowledge, self‐care and improvements in physical health indicators such as blood pressure and weight. Additionally, an 8‐week educational programme focused on health eating and physical exercise for people living with mental illness also found reductions in systolic blood pressure and body mass index (Raynor et al. 2024). The impact of our intervention as a one‐off session demonstrates that further investigation of the ‘dose’ of education required to achieve change in knowledge and the subsequent health behaviours is required.

Competence in the delivery of health education competence is fundamental to nursing practice and underpins effective patient education across clinical settings (Pueyo‐Garrigues et al. 2022). The Heart Sense initiative offered a unique opportunity for preregistration nursing students to provide blood pressure health education to people living with mental illness. Participation of nursing students in health promotion and education increases self‐esteem and deepens knowledge of health promotion (Lane‐Martin 2019). While simulation and virtual reality have been effective in preparing preregistration nurses for patient education (Blaak et al. 2025), experience in real life practice provides the opportunity to practise communication skills, acquire clinical knowledge, administer advice and build confidence (Abbott et al. 2012). Exposing students to this kind of nursing role also encourages them to explore various nursing career pathways outside the hospital setting. This has the potential to enhance retention of students in the profession as they find roles that they perceive as satisfying and fulfilling (Calma et al. 2022).

Positioning nursing students as educators aligns with experiential learning approaches, and emphasises learning through practice and clinical reflection to build health promotion capability and confidence (Patterson et al. 2017). Additionally, sustained, high quality contact with people living with mental illness has been shown to reduce nursing student stigma towards mental illness, with positive implications for the future of mental healthcare (Moxham et al. 2016; Roberts et al. 2025; Yousiph et al. 2025). To our knowledge, few studies have examined nursing students' attitudes and confidence in delivering health education within community mental health placements. The present findings begin to address this gap, suggesting that structured, authentic blood pressure education activities can be feasibly embedded within clinical placements in ways that both enhance student competence, knowledge acquisition, and consumers empowerment, while contributing to improved attitudes towards the comorbid physical health issues that people with mental illness live with.

### 5.1 Limitations

There are several limitations. As consumer participants reside in a single geographical region and nursing participants were from one university, this potentially reduces the generalizability of findings to other groups. A larger sample size of more diverse participants would increase confidence in the results, and future research on this programme will continue to add to the evidence base. The non‐traditional clinical placement environment used in this study is unique, potentially limiting the transferability of this programme to other mental health clinical placement settings. Additionally, longer follow up periods and the incorporation of other outcome measures, such as changes in health behaviour, would provide a more detailed evaluation of the interventions' impact.

## 6 Conclusion

Heart Sense demonstrates the potential value of embedding a brief, supervised blood pressure education activity within a community mental health clinical placement. This study demonstrated improved consumer hypertension knowledge following the student led intervention. In addition, nursing students reported increased competence across health education knowledge, skills and attitudes. This dual impact supports integrating structured blood pressure education into mental health placements as a practical way to address cardiovascular risk inequities while strengthening students' capacity to deliver person centred education. Future research should investigate longer‐term outcomes, replication across diverse community settings and whether improved consumer knowledge translates into sustained self‐management behaviors and measurable improvements in cardiovascular risk factors.

### 6.1 Relevance for Clinical Practice

This study demonstrates that authentic, student led blood pressure education for people with lived experience of mental illness significantly enhances preregistration nursing students' confidence and competence in health promotion, addressing a critical gap in experiential learning within nursing curricula. For nursing education, these findings advocate embedding real world practice to better prepare students for their foundational health educator role as registered nurses. In clinical practice, the intervention model supports the upskilling of nursing students to deliver targeted hypertension education, tackling cardiometabolic disparities while fostering consumer knowledge for at risk populations.

## Author Contributions

L.M., C.S., C.P.: conceptualization; E.H., C.S., T.Y., E.‐K.J.: data curation; C.S., B.S., E.H.: formal analysis; C.S., L.M., C.P., T.Y., E.H.: funding acquisition; C.S., T.Y., B.S., E.‐K.J., L.M., C.P.: investigation; C.S., T.Y., L.M., C.P.: methodology; C.S.: project administration; C.S., T.Y., B.S., L.M., C.P., E.C., J.M.: resources; C.S., T.Y., E.H.: software; L.M., C.P., E.H.: supervision; C.S., E.H.: visualisation; C.S., T.Y., E.H., C.P., L.M.: writing – original draft; C.S., T.Y., E.H., B.S., C.P., E.C., J.M., L.M.: writing – review and editing.

## Funding

This study was supported by the University of Wollongong Health Innovations EMCR Seed Grant. The funder provided financial support to undertake the research.

## Ethics Statement

University of Wollongong Human Research Ethics Committee (HREC), Australia Approval No H2025‐1462.

## Conflicts of Interest

Recovery Camp is a research and social impact program that has been supported by the University of Wollongong to explore its potential as a social enterprise. Authors C. Patterson and L. Moxham are directors of Recovery Camp Pty. Ltd.

## Data Availability

The data that support the findings of this study are available on request from the corresponding author. The data are not publicly available due to privacy or ethical restrictions.
